# Are Mobility and COVID-19 Related? A Dynamic Analysis for Portuguese Districts

**DOI:** 10.3390/e23060786

**Published:** 2021-06-21

**Authors:** António Casa Nova, Paulo Ferreira, Dora Almeida, Andreia Dionísio, Derick Quintino

**Affiliations:** 1 Instituto Politécnico de Portalegre, 7300-110 Portalegre, Portugal; casanova@ipportalegre.pt; 2 VALORIZA—Research Center for Endogenous Resource Valorization, 7300-555 Portalegre, Portugal; 3 CEFAGE-UE, IIFA, Universidade de Évora, Largo dos Colegiais 2, 7004-516 Évora, Portugal; dmfa1982@gmail.com (D.A.); andreia@uevora.pt (A.D.); 4 Department of Economics, Administration and Sociology, University of São Paulo, Piracicaba 13418-900, SP, Brazil; derickdq@usp.br

**Keywords:** correlation coefficient, detrended cross-correlation analysis, COVID-19, mobility indices

## Abstract

In this research work, we propose to assess the dynamic correlation between different mobility indices, measured on a daily basis, and the new cases of COVID-19 in the different Portuguese districts. The analysis is based on global correlation measures, which capture linear and non-linear relationships in time series, in a robust and dynamic way, in a period without significant changes of non-pharmacological measures. The results show that mobility in retail and recreation, grocery and pharmacy, and public transport shows a higher correlation with new COVID-19 cases than mobility in parks, workplaces or residences. It should also be noted that this relationship is lower in districts with lower population density, which leads to the need for differentiated confinement policies in order to minimize the impacts of a terrible economic and social crisis.

## 1. Introduction

The numbers of COVID-19 cases, both infections and casualties, are increasing daily all over the world, and concerns about their effects show no decrease. Even with the start of vaccination programs, it has not been possible to break the advance of the numbers, primarily because the speed of vaccination is asymmetric in different countries, but also because, contrarily to some respiratory diseases in the past, the spread between countries was higher [1,2,3]. With various negative economic and financial effects (see References [4,5,6,7,8,9,10]), COVID-19 also has several other consequences in people’s lives, such as fear and depression [11,12], suicide trends [13] or in mental health [14,15].

The substantial effects of COVID-19 are related to the lockdowns that countries had to impose to control the spread of the disease. According to Reference [16], human behavior, among other factors, could contribute to respiratory viral infections, even more in a context where the superspreading conditions are not fully known [17]. However, it is crucial to reduce the number of social contacts, as complete vaccination programs are absent or not yet fully developed, and social-distancing measures could be the key in helping to solve the problem [18].

The spread of COVID-19 could be related to several factors. For example, Reference [19] identified several of these factors in assessing community risk factors in Catalonia, Spain, such as air pollution, population density, demographic and socioeconomic conditions, or even land use. In addition to these factors, which could affect the incidence of the disease in a general way, the authors also identify other factors related to the possible individual prevalence of the disease, such as the existence of comorbidities.

The existence of social contacts could be proxied by mobility data [20], with frameworks such as Google’s Community Mobility Reports (CMR) being able to measure that mobility, as it measures citizens’ mobility according to different types (for more details about CMR, see References [21,22]).

The use of CMR and its effects in COVID-19 has already been made using different approaches; see, for example, the studies of References [20,23,24,25,26,27,28], which, at a country level, found that the reduction of the mobility has a direct impact on the decrease of the infections. Reference [29] also confirms these trends and adds that reducing cases due to mobility restrictions has a very significant effect on a 2-week basis.

At a regional level, we can find the studies of References [30,31], both for the US. Although both find relevance in the effect of mobility on controlling the disease, Reference [30] finds differences between urban and rural locations, while Reference [31] identifies that population density has different implications in the reduction of mobility (higher density has more impact on the reduction of mobility, for example, in stores). In Poland, Reference [32] concluded that the restrictions helped control COVID-19, although with the difference between regions, related to the strictness of state restrictions.

During January 2021, Portugal was constantly in the news, as it was considered the worst country in the world regarding the infections and death rate (see https://www.politico.eu/article/portugal-coronavirus-rate-surge/, accessed on 19 May 2021). The lifting of some restrictions during the Christmas season may have compounded this tragic scenario. In this context, our purpose is to analyze, in a dynamic way, and based on daily data, the relationship between citizens’ mobility and new COVID-19 infections, using regional-level data, in this case, for Portuguese districts. Our main objective is to assess the relevant relationship between the number of new infections of COVID-19 and citizen mobility. Moreover, we also want to distinguish between the different types of mobility. Differentiating the analysis between regions could give important insights for possible future decisions about new lockdowns or lifting of restrictions.

The implementation of non-pharmacological measures has a relevant impact on the control of the dissemination of COVID-19. In Portugal, the introduction of mandatory personal protective equipment (PPE) such as masks, or the instructions for frequent use of alcohol gel and washing hands, among others, started with the beginning of the pandemic in March/April 2020. Since then, the use of PPE has remained mandatory, and the non-pharmacological measures have not changed significantly.

In this paper, the mobility is measured considering Google CMR reports, and the relationship between mobility and new cases is assessed through the detrended cross-correlation analysis correlation coefficient. This non-linear framework has the ability to capture the relationship between variables for different timescales, which could give important information about the number of days needed to reduce infections. Moreover, we also propose the use of a sliding windows approach, which allows analysis of the evolution of the relationship over time.

Our main results corroborate that mobility is correlated with the number of new COVID-19 cases. However, the mobility correlation is not equal for the different typologies: for example, mobility in retail, recreation and groceries seems to have a higher correlation, while in general the mobility in workplaces shows little relationship. Despite the temporal evolution of the relationship, confirming that the lift of restrictions at Christmas had a highly significant impact on the increase of new COVID-19 cases, we also find that the impacts of the mobility are different across districts.

The remainder of the paper is organized as follows: in Section 2, both data and methodology are presented, with the results being present in Section 3, while Section 4 provides discussion and conclusions for the study.

## 2. Data and Methodology

Since the outbreak of COVID-19, and until 13 April 2021, almost 138 million cases were reported worldwide, with almost 3 million deaths. Portugal has about 828,000 cases and around 17,000 deaths. For cases of disease, information is available from the Portuguese Health Ministry, through Sistema Nacional de Vigilância Epidemiológica (SINAVE), with the complete set of registered cases until 28 February 2021 (due to data availability). Until this day, Portugal has had a total of 805,140 cases. Intending to analyze the relationship between mobility and COVID-19 in the different Portuguese districts, we considered only the information which is registered in Portuguese mainland districts due to the availability of data about mobility. In total, the number of cases of the districts is 775,954. All the data were transformed in daily incidence for each district to perform the correlational analysis with the information from Google CMR. In these reports, it is possible to retrieve information about six distinct mobility indices: (i) retail and recreation (I1); (ii) groceries and pharmacies (I2), (iii) parks (I3), (iv) transit stations (I4), (v) workplaces (I5) and (vi) residential areas (I6). For more information about the indices and the places where mobility is referred to, see https://www.google.com/covid19/mobility/index.html?hl=en (accessed on 19 May 2021).

Daily data for these indices were retrieved for Portuguese districts from 15 February 2020 to 28 February 2021, in a total of 380 observations. Some districts do not have information for the mobility indices in some days of August and September 2020, implying that the sample is smaller for those districts (355 observations). The information about the number of cases and the number of observations for each district are identified in Table 1. Moreover, as some districts present missing information for some indices, the correlations were calculated for the remainder, where data are available.

To perform our correlational analysis, we use the detrended cross-correlation analysis coefficient (ρDCCA), proposed by Reference [33] and derived from the work of Reference [34]. The DCCA measures the long-range cross-correlation between two series $Y_{i}$ and $X_{i}$ consisting on the sequence of k=1,2,… ,N observations. The first step of the DCCA consists of the calculation of the profiles:

(1) $$Y_{k}=\sum_{i=1}^{k}\left(y_{i}-\langle y\rangle \right)\;\mathrm{and}\;X_{k}=\sum_{i=1}^{k}\left(x_{i}-\langle x\rangle \right)$$

with 〈.〉 as the mean operator. Those profiles are then divided into (N−n) overlapping boxes, from n=4 to n=N/4 and for each box, based on the ordinary least squares, local trends ${\tilde{Y}}_{k,i}$ and ${\tilde{X}}_{k,i}$ are calculated, for future detrend of the profiles $Y_{k}$ and $X_{k}$. With the local trends, the covariance of the residuals of each box is calculated as follows:

(2) $$f_{xy}^{2}\left(n,i\right)=\frac{1}{\left(n+1\right)}\sum_{k=1}^{i+n}\left(X_{k}-{\tilde{X}}_{k,i}\right)\left(Y_{k}-{\tilde{Y}}_{k,i}\right).$$

Considering the information of all the set of N−n boxes, the DCCA covariance is calculated as follows:

(3) $$F_{xy}^{2}\left(n\right)=\frac{1}{\left(N-n\right)}\sum_{i=1}^{N-n}f_{xy}^{2}\left(n,i\right),$$

which was used by Reference [33] to obtain the correlation coefficient given by the following:

(4) $$\rho_{DCCA}=\frac{F_{xy}^{2}\left(n\right)}{F_{x}^{2}\left(n\right)F_{y}^{2}\left(n\right)}.$$

The denominator of $\rho_{DCCA}$ consists of the fluctuation functions of the detrended fluctuation analysis of Reference [35], which analyzes the long-range behavior of each time series individually.

The $\rho_{DCCA}$ is a non-linear correlation coefficient, robust to the presence of non-stationarity, and confirms the property of $-1\leq \rho_{DCCA}\leq 1$ according to [36,37,38,39] and is testable according to [40]. Moreover, this is a multiscale correlation coefficient, allowing for the analysis of the behavior between variables in different time periods. Despite the statistical properties previously referred to, the robustness of the correlation coefficient is confirmed by its use in different research areas (see, for example, [41,42,43,44,45,46], among others).

In this analysis, the *ρ_DCCA_* will be calculated using a sliding windows approach to analyze the evolution of the correlation over time, using windows of 250 observations. In Table 2 we present the critical values to test the null hypothesis of absence of correlation, considering 250 observations, as it is the dimension of the samples used in the analysis.

## 3. Results

As previously stated, this study uses the DCCA correlation coefficient to assess the relationship between mobility indices and COVID-19 in Portuguese districts, also applying a sliding windows approach in order to evaluate the evolution of the correlation over time.

**Figure 1:**
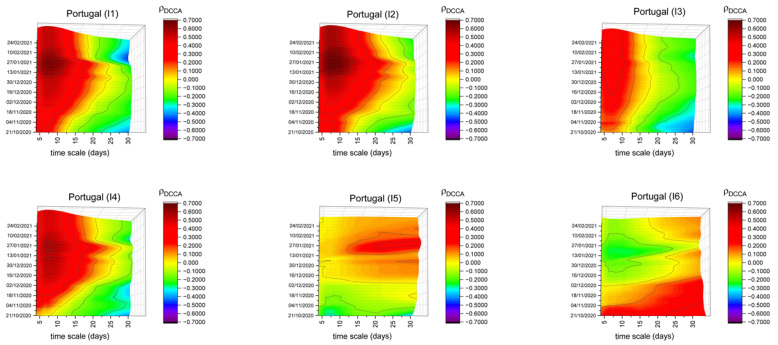
DCCA correlation coefficients between the different mobility indices and new COVID-19 cases in Portugal.

**Figure A1:**
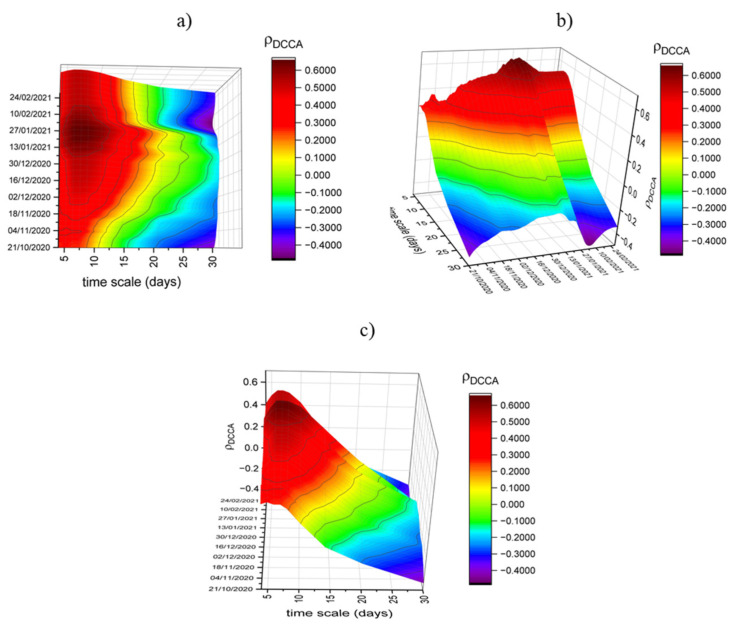
Representation of the DCCA correlation coefficient between the retail and recreation mobility index and new COVID-19 cases in Portugal. Panel (**a**) reinforces the analysis through the time-scale view; panel (**b**) reinforces the analysis through the view of the temporal evolution; panel (**c**) shows a panoramic view of the results.

Figure 1 shows the behavior of the DCCA correlation coefficient between new COVID-19 cases and the six mobility indices, identifying the evolution over time for Portugal as a whole. Considering the multiscale feature of the measure and the temporal dynamics, a tri-dimensional analysis could be made. The information could be represented in different dimensions, as we can see in Figure A1, Appendix A. There, the results for Portugal as a whole are available, considering the correlation between the retail and recreation index and new COVID-19 cases, in three panels. Panel (a) reinforces the difference between time scales; in panel (b), the view is more about the evolution of the correlation over time; panel (c) adopts a panoramic view and is the one chosen for presentation the general results throughout the paper.

The results may be analyzed through different dimensions and perspectives, allowing an in-depth interpretation of the results.

Firstly, in general, the behavior of the correlation of retail and recreation, groceries and pharmacies and transit stations indices is qualitatively similar. In the very short run (lower timescales) the correlation coefficients are relatively high, meaning that mobility has a positive correlation with the number of new cases. However, there is a time-varying behavior, with a significant increase at the beginning of 2021, more marked in the case of groceries and pharmacies. Despite the continuous increase in the correlation, a peak can clearly be noticed after Christmas, probably related to the lifting of mobility restrictions in the country (in a season when environmental conditions could be more conducive to the development of respiratory problems). In the middle of January 2021, the Portuguese government took severe restrictive measures. Immediately afterwards, the correlation levels remained high, meaning that the mandatory restrictions to the mobility probably had a significant correlation with the reduction in the number of new COVID-19 cases. Over time, those measures could have had result on a progressive decline of the correlation levels, in agreement with References [20,23,24,25,26,27,28]. Another important feature is that the peak of the correlation is about the 7th/8th day, although in the groceries and pharmacies index it seems to be a little bit more, but it is remarkable that the duration of the correlations (red ones) is higher during the peak of the beginning of 2021. This means that lifting mobility restrictions or imposing new mobility restrictions could have an expected impact in about a week, which is consistent, for example, with the incubation period of the virus [47,48,49].

The results of the correlation of parks mobility index show different behavior and are more constant over time. Even though it seems relevant to explain the increase of new COVID-19 cases, the impact of this mobility type is not as high. As it measures mobility in open spaces, it should be related to a lower capacity of contagion in those spaces.

Finally, the correlation of the workplaces and residential areas indices presents different behavior, also considering the differences of the places to which they refer. Compared with the previously analyzed indices, the reduced correlations in workplaces mean that they seem to be relatively secure locals, probably due to the different measures taken by the employers. Despite the reduced levels of the correlations compared with the previously analyzed indices, the workplaces index seems to increase its correlation with COVID-19 cases over time during part of the sample, moving from negative to positive correlations in mid-December and continuing to increase during January and February. Moreover, it is important to highlight that at the beginning of 2021, the correlation is higher for higher timescales.

The results of the correlation of the workplaces index could firstly be justified with a period of a greater confluence of employees to their workplaces, especially before Christmas and New Year and, after this, the sharper increase may reflect the lifting of measures to restrict mobility during the Christmas and New Year period. Workplaces concern with the active population, that is, mostly between 30 and 50 years old. It is in this age group that asymptomatic cases are most significant. So, it could be a “domino effect”: people left for Christmas, the “family bubbles” were broken, and when they returned to work, they infected others, which could justify the increased correlation and the impact even in longer timescales.

Regarding mobility in residential areas, as expected, it has higher moments of negative correlations, meaning that keeping people in their homes would decrease new infections. This finding is similar to References [30,50], both for the case of the US. However, it is noteworthy that some positive correlations are noted at the beginning of the analysis, although weaker than in the other mobility indices. This could happen because during the first months of the pandemic, most disease cases could have appeared in family circles.

As a final note referring to the statistical significance of the correlations, due to the multidimensional analysis, it is not feasible to introduce the information of the critical values in the figures. For this, it is necessary to identify the critical values from Table 2. Roughly, it is possible to say that, until n = 16, orange plans mean statistical significance, while, for higher timescales, darker oranges or blue plans are necessary.

**Figure A2:**
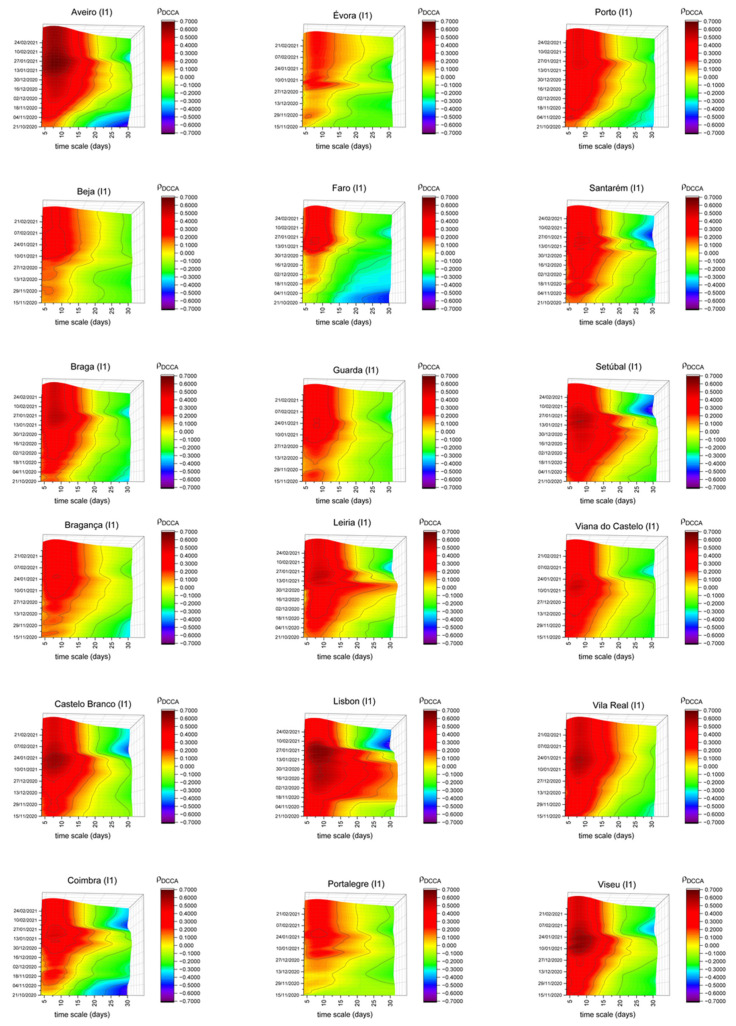
DCCA correlation coefficients between the retail and recreation index and new COVID-19 cases in the complete set of Portuguese districts.

**Figure A3:**
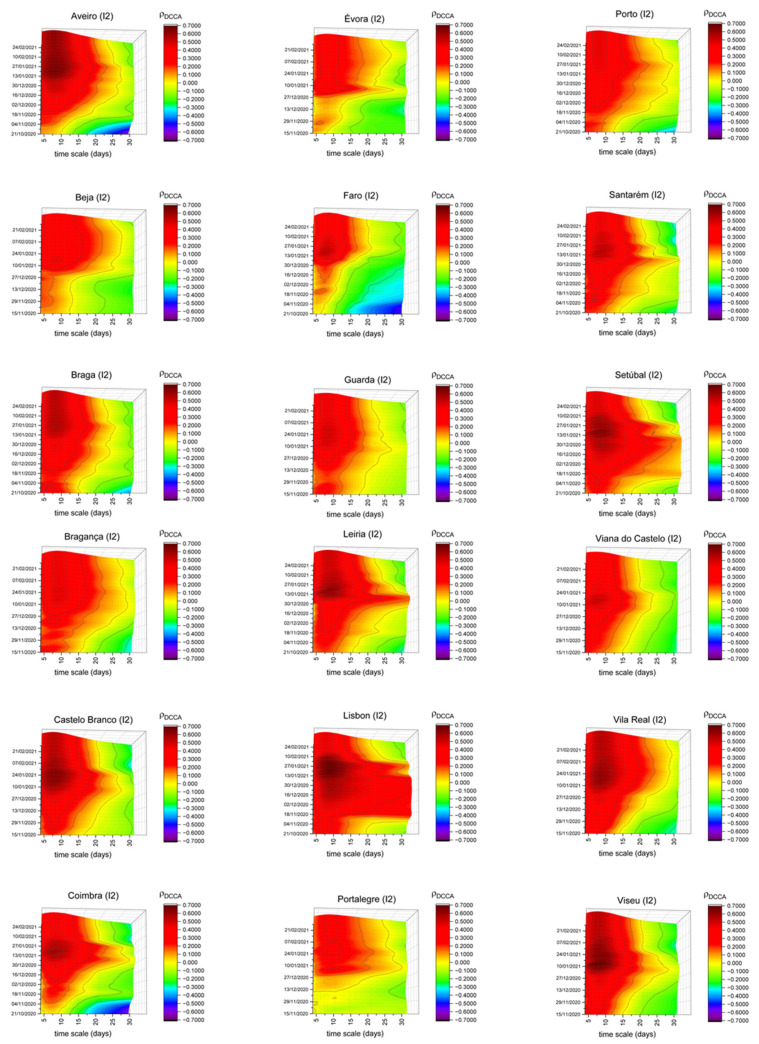
DCCA correlation coefficients between the groceries and pharmacies index and new COVID-19 cases in the complete set of Portuguese districts.

**Figure A4:**
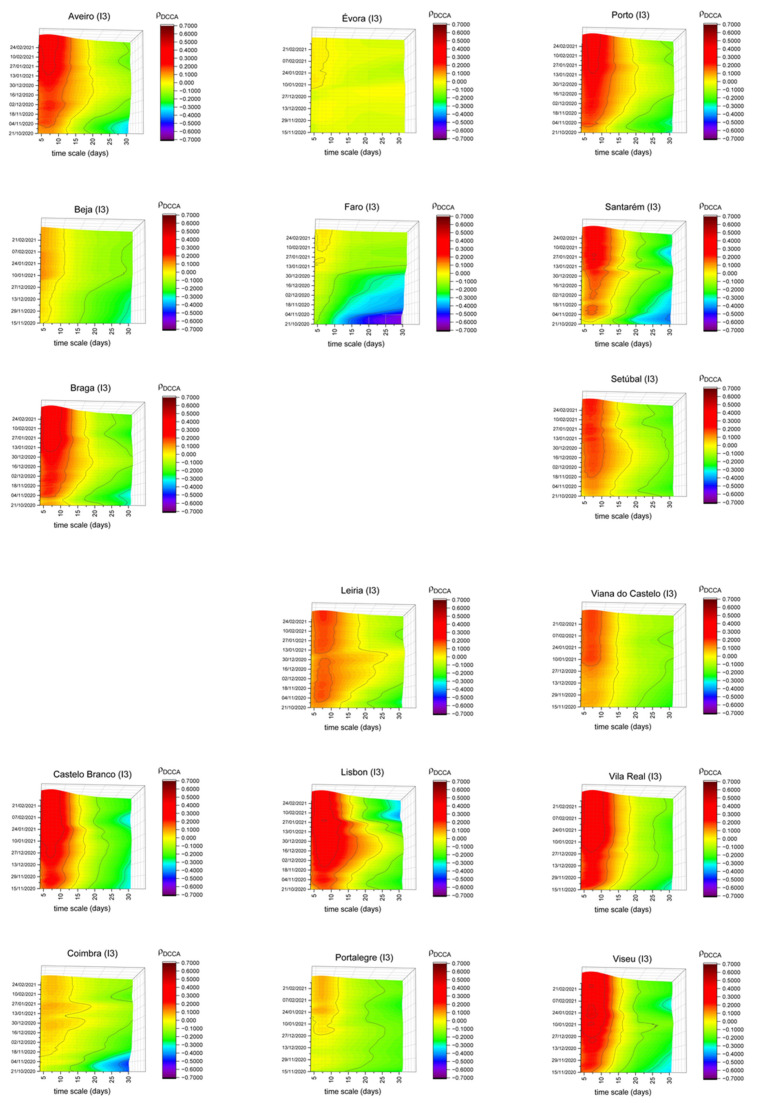
DCCA correlation coefficients between the parks index and new COVID-19 cases in the complete set of Portuguese districts.

**Figure A5:**
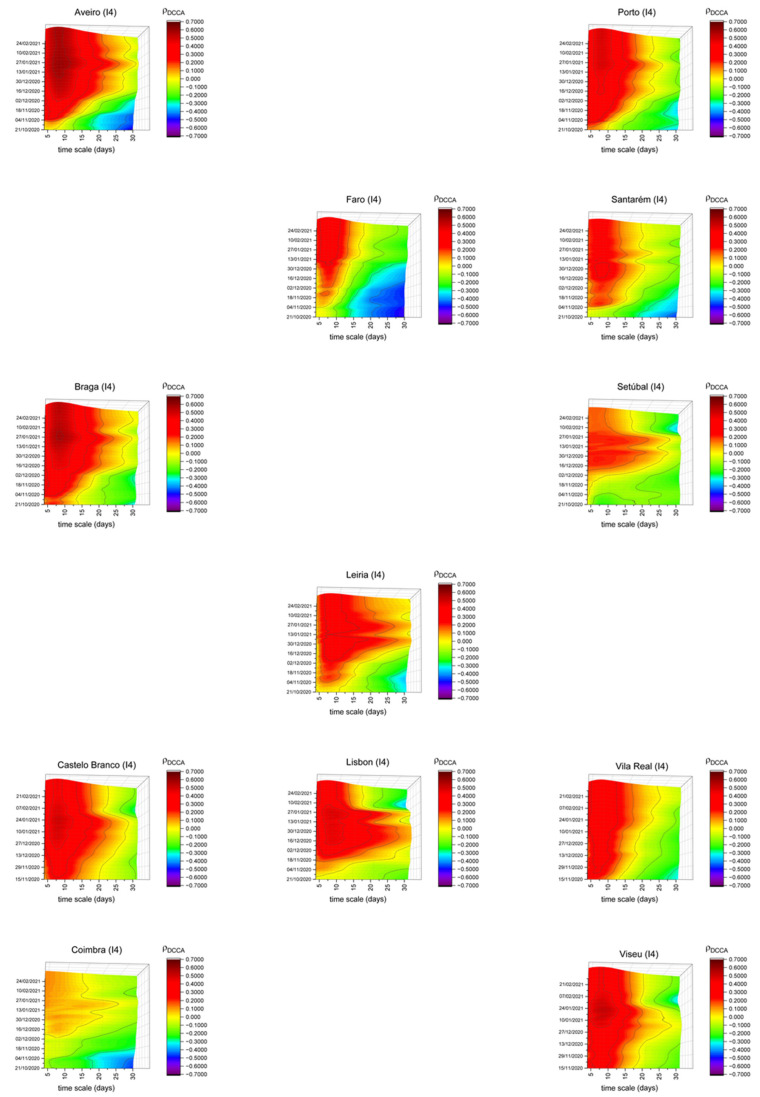
DCCA correlation coefficients between the transit stations index and new COVID-19 cases in the complete set of Portuguese districts.

**Figure A6:**
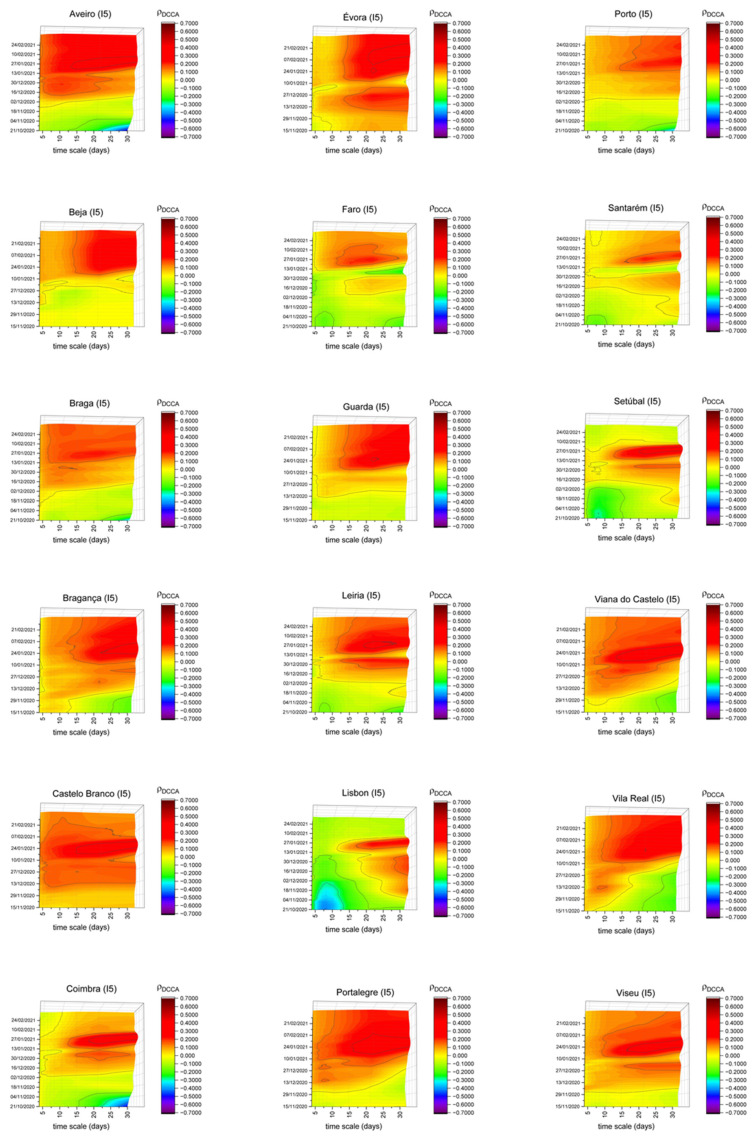
DCCA correlation coefficients between the workplaces index and new COVID-19 cases in the complete set of Portuguese districts.

**Figure A7:**
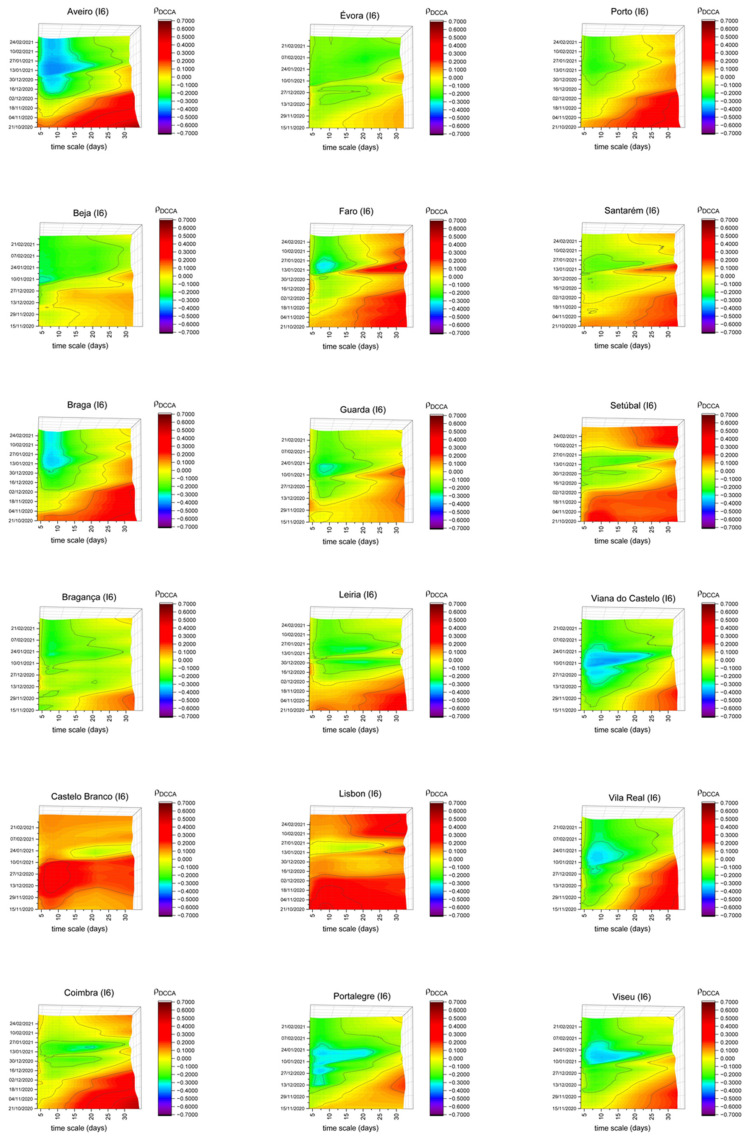
DCCA correlation coefficients between the residential areas index and new COVID-19 cases in the complete set of Portuguese districts.

In addition to the global analysis, we also aimed to analyze the relationship between mobility and COVID-19 in the different Portuguese districts. To do that, we made a similar analysis for each district, comparing it with the results presented for Portugal as a whole. Due to space limitation, we highlight non-similar patterns on the analysis of those indices (all the figures, organized by indices, are presented in Appendix B, in Figure A2, Figure A3, Figure A4, Figure A5, Figure A6 and Figure A7. The existence of significant differences across districts could lead to thinking that adopting different lockdown measures between districts should be a hypothesis to be considered.

**Figure 2:**
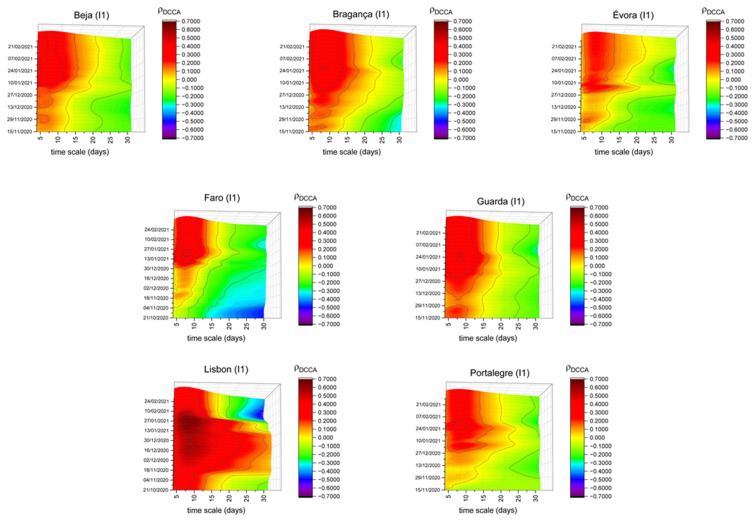
DCCA correlation coefficients between retail and recreation (I1) and new COVID-19 cases in Beja, Bragança, Évora, Faro, Guarda, Lisbon and Portalegre.

If we consider the retail and recreation index (I1) (see Figure A2), in general, all districts in the country show a similar correlation pattern with the national results. This pattern is characterized by a lower correlation at the beginning of the sample period, increasing gradually until its peak at the beginning of 2021. Despite this similar pattern, it is important to mention districts such as Beja, Bragança, Évora, Faro, Guarda and Portalegre, in which the correlation intensity is lower than that found for Portugal, as seen in Figure 2. Excepting Faro, these districts are located in inland (and more rural) regions which have lower population density levels, in line with Reference [30]. Another district that we consider relevant to include is the Lisbon district. Between mid-November and early February, high correlation levels are observed for the different timescales. This evidence contrasts with that observed at the national level, which shows higher correlation levels for the same period, mainly for short timescales. This behavior may reflect the greater confluence of people in this type of space, not only in the period leading up to Christmas and New Year (for the traditional festive season shopping) but also in the period that followed (taking advantage, for example, of the traditional sales season). The fact that high levels of correlation are observed for longer timescales may indicate the need for restrictive measures to be adopted earlier.

These features lead us to think about the possibility of dichotomies between inland and coast, which could allow the conclusion that mobility restrictions could have been differentiated according to these dichotomies.

**Figure 3:**
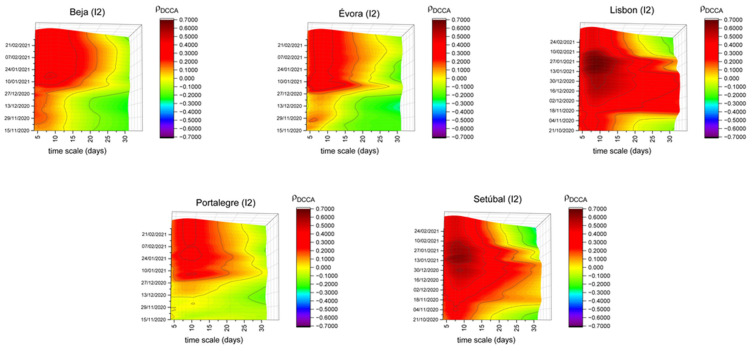
DCCA correlation coefficients between groceries and pharmacies (I2) and new COVID-19 cases in Beja, Évora, Lisbon, Portalegre and Setúbal.

Figure 3 shows the correlation patterns between the groceries and pharmacies (I2) mobility index and new COVID-19 cases for Beja, Évora, Lisbon, Portalegre and Setúbal. For Beja, Évora and Portalegre, until nearly the end of 2020, this index presented a low correlation, close to zero, lower than that found for Portugal, indicating a lesser correlation between this type of mobility on the number of new cases for these districts. This empirical evidence may be justified by the smaller number of spaces available in these districts, which are still sufficiently available to serve the needs of their populations. From the beginning of 2021 and for short timescales, these correlations have increased, which may show that the frequency of these spaces could have a positive correlation with the emergence of new cases. This evidence may reflect the return home at the end of the holiday season and the onset of symptoms. These are all inland districts, with lower population density levels, as already stated, which reinforces the possibility of the adoption of differentiated confinement measures.

Regarding Lisbon and Setúbal, between mid-November and early February, high correlation levels are observed for the different timescales. Contrary to that stated for the Beja, Évora and Portalegre districts, Lisbon and Setúbal are districts with high population density, which may justify the observed behavior. Furthermore, it could also be justified not only by the high number of this kind of space but also by the increase in the number of people who go to those places. This could lead us to think that the adoption of different measures (more restrictive in this case) should be considered.

**Figure 4:**
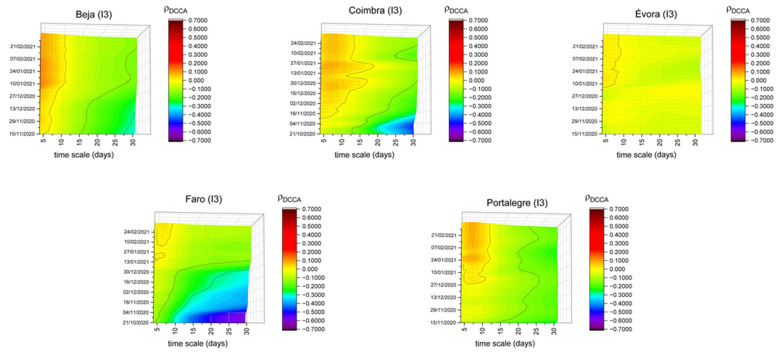
DCCA correlation coefficients between parks (I3) and new COVID-19 cases in Beja, Coimbra, Évora, Faro and Portalegre.

In Figure 4, we have selected Beja, Coimbra, Évora, Faro and Portalegre because they present a different pattern compared to the parks index presented in Figure 1. This index has a lower correlation with the number of new cases, when compared to those found for Portugal. Parks refers to open spaces, where it is known that the propagation of the virus could be less significant. The low population density could also explain the differences of Beja, Évora and Portalegre, as was found by Reference [51] for US counties, while Faro’s location, on the south coast of Portugal, and the extension of its beaches, could lead to different results (i.e., enjoying those type of open spaces cautiously could imply lower correlation levels). Regarding Coimbra, it is also a district that is close to beaches but also with some municipalities with reduced population density levels. It is also necessary to highlight that, in Faro, the sliding windows correlation coefficients until November show high negative values, meaning that the possibility of enjoying time in those open spaces was negatively correlated with the increase of new COVID-19 cases.

On the other hand, in the period following the adoption of the new confinement measures, an increase could be noted in the correlation between this index and the number of new cases, which may reflect the possibility of using the so-called “hygienic walks”. Thus, the adoption of restrictive measures concerning the frequency of use of these spaces may seem counterproductive. In other words, the fact that some of these spaces closed completely (e.g., walled public gardens), may have led to the displacement of people to those where only circulation was allowed (and not staying there), having an impact on the increase in correlation, especially on short timescales.

Before we start our analysis about the transit stations (I4) index for some districts, we would like to state that this is the only index for which some districts do not have available information, which may be related to lesser presence of public transportation.

**Figure 5:**
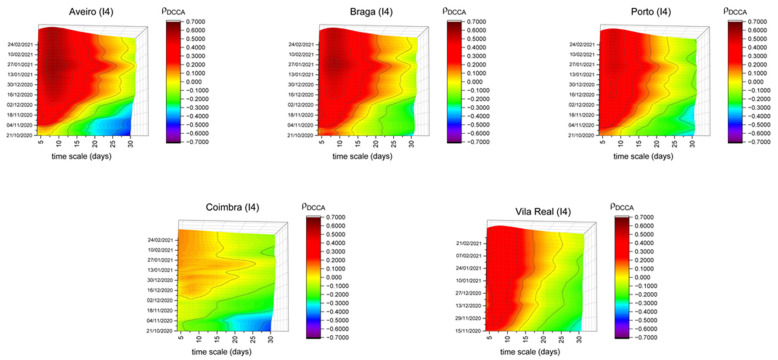
DCCA correlation coefficients between the mobility in transit stations (I4) and new COVID-19 cases in Aveiro, Braga, Porto, Coimbra and Vila Real.

Figure 5 shows the correlation between the indices referring to the mobility in transit stations and new COVID-19 cases for five different districts, all located in the north region. In mid-January, new confinement measures were adopted by the government. They had a national impact, which could have led to the reduction of the correlation between this index and the number of new COVID-19 cases; however, there was no significant correlation reduction in Aveiro, Braga and Porto. This mobility index continued to show high correlations for short timescales with the number of new COVID-19 cases. Regarding Coimbra, its correlation is lower over the entire sample period and for all timescales. On the one hand, it may indicate the security of the transport network or a lower rate of its usage in this district. Finally, in Vila Real, we can see higher correlations in the short-term, without significant change over time. This fact may reflect that transport habits in this district have remained unchanged.

**Figure 6:**
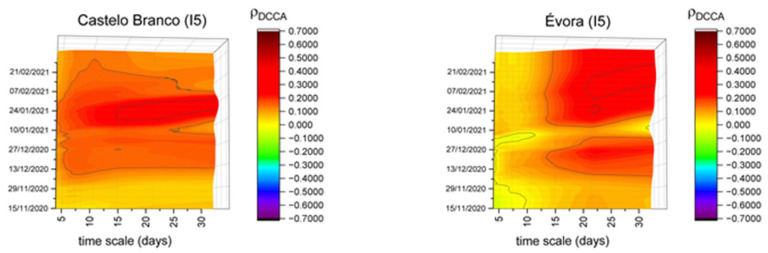
DCCA correlation coefficients between workplace mobility (I5) and new COVID-19 cases in Castelo Branco and Évora.

Comparing the results in workplace mobility (I5), it is possible to distinguish a different pattern of correlations mainly in Évora and Castelo Branco, as represented in Figure 6. These are the only districts with significant differences throughout the period under analysis for the different timescales, showing a positive correlation between this index and new COVID-19 cases. This could be related to less efficient security measures in workplaces or the fact that they were adopted later.

**Figure 7:**
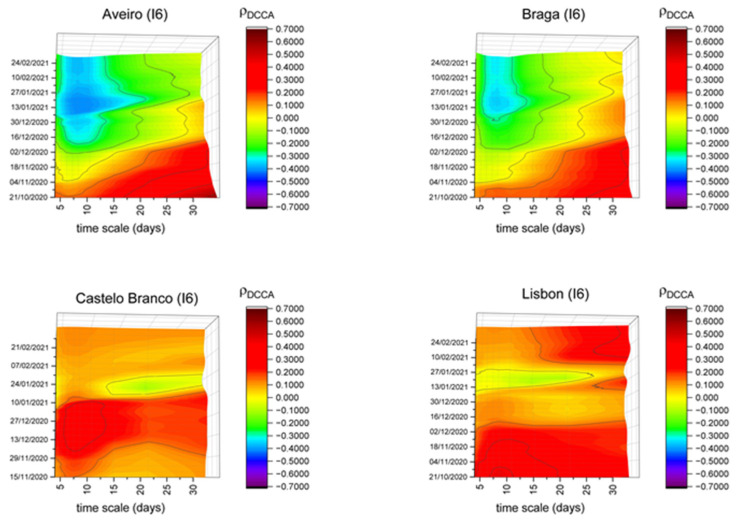
DCCA correlation coefficients between the residential areas (I6) index and new COVID-19 cases in Aveiro, Braga, Castelo Branco and Lisbon.

Finally, considering the residential areas (I6) index, Figure 7 shows the patterns registered in Aveiro, Braga, Castelo Branco and Lisbon, although with different patterns. Aveiro and Braga show the highest negative correlations after the confinement of the beginning of 2021, probably meaning that the success of the lockdown was greater in those districts. Regarding Castelo Branco and Lisbon, these districts are the only ones showing a positive correlation over the entire sample period, mainly in short timescales. This may indicate that, in these districts, the family nuclei could have caused an emergence of new COVID-19 cases, although with different possible explanations. Lisbon is the most populous district of the country and in some cases the quality or undersized dimensions of the habitations could promote the increase of contagion. On the other hand, in the case of Castelo Branco, the situation could be related to an existent gap between the beginning of the cases in this district and the rest of the country. For example, when the first confinement occurred, Castelo Branco had practically no COVID-19 cases, meaning that people had no necessity to go to their houses, i.e., confinement could be considered unnecessary in the district.

Taking the different patterns found for the correlations between some of the six indices and the spread of new COVID-19 cases, we would like to highlight that confinement measures do not have the same effect on all districts, which could indicate that the adoption of different measures in different districts could be desirable. We also highlight that there are locations that seem to present more risk of contagion (the ones related to retail and recreation, groceries and pharmacies and transit stations), while residential areas seem to present a lower risk factor of contagion, as expected. Applying the DCCA coefficient, an unexplored method to address this issue, allows us to analyze the behavior between each mobility index and the spread of new COVID-19 cases in different timescales and leads us to understand, for example, when peak correlations occurred and that not all the indices have the same peak correlation.

## 4. Discussion and Conclusions

In this research work, the intention was to assess the correlation between the number of contagions and the mobility indices of people. For this purpose, an approach based on the DCCA was used, which has the capacity to assess the global correlation between serial variables. Simultaneously, it presents robustness in the face of issues related to stationarity, non-linearity and non-normality of the data and also allows for the analysis of the evolution of the relationship over time. The whole sample under consideration was Portugal and its respective districts, with daily data on the variables under analysis. It should be noted that, despite Portugal being an interesting case study, as it was considered exemplary in the first phase of the pandemic crisis and was catalogued as the “worst country in the world” in January 2021, the truth is that the approach is robust and valid and can be successfully applied to any country or region.

The global results essentially indicate that a dynamic association exists between the different mobility indices and the new COVID-19 cases, with three main risk factors being identified in terms of mobility: retail and recreation (I1); groceries and pharmacies (I2) and public transport (I4). In addition to the considerations already taken, regarding the effectiveness of confinement to contain contagions, we can infer that some of these mobility factors may imply the non-use of a mask in certain situations, which may justify the values found for the retail and recreation and groceries and pharmacies indices. Take as an example recreation (cafes and restaurants) in which the consumption of food and drink goods prevents the use of a mask. In the case of public transport (I4), it could also be related to the fact that people may touch the same surfaces sequentially, with the respective risk of contagion.

When we perform the district analysis, for the majority of districts, we found similar behavior to that of the country as a whole. However, there are some distinct behaviors during the period under analysis and for different mobility risk factors. These differences may be related to the low population densities of some districts, especially those inland. Note that, for all the indices except residential areas, in general, the least densely populated districts were the ones showing lower correlations than those of the country as a whole, in line with the results found in Reference [31]. Regarding residential areas, Lisbon has a higher level of correlation than the average for Portugal, which may indicate that, in large cities, with a high population density and possibly weaker habitational conditions, residential mobility may be a significant contagion factor. Once again, it is the districts with the lowest population density that stand out (due to the lowest correlation) in this factor, also related to the difference between urban and rural areas, as identified by Reference [30].

Overall, and always bearing in mind that other factors could be related to the increasing number of new COVID-19 cases, as stated by Reference [19], we can conclude that some mobility indices are more likely than others to have correlation patterns with the contagion levels of COVID-19, which may be linked to the crowding of people, wearing masks and hand hygiene. In addition to this, we also concluded that population density might affect the correlation level of mobility indices with the new confirmed cases of COVID-19. It appears that districts with lower population density have lower correlations, which indicates that a different definition of confinement policies may be more appropriate for controlling the pandemic and simultaneously minimizing its effects in economic and social terms. Blindly imposing confinements leads to population revolt and the growth of states of anxiety and general impoverishment. It is increasingly important to understand which risk factors related to mobility most potentiate contagion and which regions and moments tougher measures are justified in terms of containment. These results are in line, for example, with the conclusions of Reference [28], where it is stated that re-arranging local restrictions can be much more effective in controlling the number of COVID-19 cases without causing unnecessary economic costs than local or country-wide mobility restrictions.

The results obtained in this study and the respective conclusions may be an important contribution to political decision-making about measures to be taken to contain the amount of contagion and, possibly taking measures which are differentiated by district and/or region, combining them with the available different non-pharmacological measures, which have been relatively stable during the period under analysis.

It is important to state again that the focus is on the method and respective abilities, which has proven to be robust and adequate, providing accurate and detailed information about the variables that have the greatest correlation with the number of COVID-19 infected persons. It is also relevant to highlight that the increased mobility in Portugal was made considering the break in social distancing, especially between family and close social meetings. Given this, we believe that there is a high probability that the increased mobility had a strong impact on the increase in numbers of people infected with COVID-19, given the tendency for breaking social distancing, especially in the Christmas period.

## Figures and Tables

**Table 1:** Total number of COVID-19 cases for each district and the number of observations considered in the analysis.

District	Total Cases	Observations	District	Total Cases	Observations
Aveiro	54,974	380	Leiria	24,647	380
Beja	7778	355	Lisbon	195,131	380
Braga	83,524	380	Portalegre	6936	355
Bragança	9787	355	Porto	160,398	380
Castelo Branco	10,914	355	Santarém	26,762	380
Coimbra	28,953	380	Setúbal	66,228	380
Évora	10,018	355	Viana do Castelo	16,920	355
Faro	19,594	380	Vila Real	13,972	355
Guarda	12,264	355	Viseu	27,154	355

**Table 2:** Critical values to test the $\rho_{DCCA}$ considering time series of 250 observations and different timescales, considering a confidence level of 95% (source: Reference [40]).

Timescale	Critical Value
*n* = 4	0.137
*n* = 8	0.152
*n* = 16	0.193
*n* = 32	0.271
*n* = 64	0.383

## Data Availability

Restrictions apply to the availability of these data. Data were obtained from Sistema Nacional de Vigilância Epidemiológica (SINAVE) and are available with the permission of Sistema Nacional de Vigilância Epidemiológica (SINAVE).
